# Optimizing Processing Techniques of Oolong Tea Balancing between High Retention of Catechins and Sensory Quality

**DOI:** 10.3390/foods12234334

**Published:** 2023-12-01

**Authors:** Xiaofeng Lu, Yanyan Lin, Yanming Tuo, Lijia Liu, Xinxin Du, Qiufang Zhu, Yunfei Hu, Yutao Shi, Liangyu Wu, Jinke Lin

**Affiliations:** College of Horticulture, Fujian Agriculture and Forestry University, Fuzhou 350002, China; lxfitea21@163.com (X.L.); 18459479520@163.com (Y.L.); tuo3152022@163.com (Y.T.); liulijia97@163.com (L.L.); duxinxin0226@163.com (X.D.); zqf216@163.com (Q.Z.); huyunfei@fafu.edu.cn (Y.H.); ytshi@wuyiu.edu.cn (Y.S.); 000q021014@fafu.edu.cn (L.W.)

**Keywords:** oolong tea processing, catechins, process optimization

## Abstract

Catechins are the major flavor substances in teas, which have a variety of health effects; however, high catechin and high sensory quality are a pair of contradictions that are difficult to coordinate. To explore the processing procedure with high catechins and high sensory quality, a single-factor processing experiment was carried out over the processing production of oolong tea. Combined with orthogonal partial least square discriminant analysis (OPLS-DA), correlation analysis, and principal component analysis (PCA), the optimal production procedure for oolong tea is as follows: red light withering for 8 h, leaf rotating for 10 min with a total standing time for 8 h, drum roasting for 5 min at 290 °C, low-temperature rolling (flattening at 4 °C for 5 min, without pressure for 1 min and under pressure for 5 min), microwave drying (800 W for 7.5 min). This study demonstrates a significant increase in the retention of catechins, which contributes to the mellow and brisk tastes of oolong tea, addressing the challenge of catechin content and sensory quality. Our study provides a novel insight into the relationship between the oolong tea processing and flavor formation.

## 1. Introduction

Catechins, including epigallocatechin (EGC), catechin (C), epicatechin (EC), epigallocatechingallate (EGCG), gallocatechingallate (GCG), epicatechingallate (ECG), and catechingallate (CG), are the major contributors to tea flavors and healthy benefits. Oolong tea is a unique semi-fermented tea in China, the total contents of catechins gradually decline over the production of oolong tea [1]. In the processing of oolong tea, catechin components will be further transformed through systematic abiotic stresses such as light, water loss, cold and heat, force, and so on [2]. Currently, most studies on the content of catechins in the initial processing of oolong tea remain at the stage of enzymatic oxidation. In the enzymatic reaction, flavonoids such as catechins undergo oxidation or polymerization to generate various flavors [3]. During oolong tea withering, the content of catechins decreases compared to fresh leaves [2]. The levels of EGCG, EGC, CG, GCG, and ECG in indoor withering are significantly higher than those in sunlight withering [4]. The fermentation of oolong tea requires fresh leaves to be constantly shaken and allowed to stand, with alternating intervals. The light shaking degree results in a higher total flavonoid content compared to the heavy shaking degree [5]. The quantity of shaking has increased, leading to an increase in the reduction of catechins [6]. The content of EC, ECG, and EGCG shows a trend of initially decreasing and then increasing during the fermentation of oolong tea [7,8]. The catechin content in oolong tea with prolonged fermentation is lower than in short fermentation [9]. The contents of catechin decrease gradually from the bud to the fourth leaf [10]. The content of catechins in oolong tea gradually decreases with an increase in the degree of roasting [11]. During long-term storage of oolong tea, the content of non-gallated catechins gradually decreases, while the content of gallated catechins remains relatively stable [12].

There are also studies on the changes in individual catechins in other tea categories. The sensory quality of green tea is primarily influenced by EGCG, EGC, and amino acids. The total catechin content in green tea in the drum roasting processing was significantly higher than that in fresh leaves, with the EGCG and ECG contents being approximately twice as high as those in fresh leaves [13]. The sensory quality of black tea is primarily influenced by tea polyphenols, particularly EGCG and ECG [14]. To improve the quality of black tea, the withering time is prolonged to promote the activities of polyphenol oxidase (PPO) and peroxidase (POD), thereby accelerating the transformation of catechins into theaflavins [15]. In different grades of the same type of white tea, the higher the EGCG content, the higher the quality grade [16]. With the increase in withering time, the total catechin content of white tea will decrease [17,18]. Currently, there have been numerous reports on the study of catechin content in different tea categories adjusted by a single process with varying parameters [19,20]. However, few reports exist on the retention rate of various catechin components during oolong tea system processing technology [21]. The study of processing methods for retaining catechins lacks comprehensiveness. There is still a lack of comprehensive understanding regarding tea with a high content of catechins obtained through processing technology. Therefore, studying the systematic processing technology of oolong tea rich in catechins is of great significance for the development of the oolong tea industry.

## 2. Materials and Methods

### 2.1. Chemical Reagents and Experimental Instruments

Glacial acetic acid (AR), disodium ethylenediamine tetraacetate (AR), and ascorbic acid (AR) were purchased from Macklin Chemical Factory (Shanghai, China). Methanol (HPLC) and standard samples were purchased from Sigma-Aldrich Co. (St. Louis, MO, USA), including gallic acid (GA, ≥98%), epigallocatechin (EGC, ≥98%), catechin (C, ≥98%), epicatechin (EC, ≥98%), epigallocatechin gallate (EGCG, ≥98%), gallocatechin-3-gallate (GCG, ≥98%), epicatechin-3-gallate (ECG, ≥98%), and catechin gallate (CG, ≥98%). Acetonitrile (HPLC) was purchased from Merck, Darmstadt, Germany.

### 2.2. Experimental Materials and Oolong Tea Processing

The material used in this study was CFT-6 cultivar grown in a tea garden of in Zhao’an County, Zhangzhou City, Fujian Province (23°42′44″ N; 117°11′39″ E). The banjhi tea shoots composed of one bud and two or three leaves were collected and applied to the production of oolong tea in this study.

The production process of oolong tea was as follows: the harvested tea shoots were subjected to white light withering (WL) for 2 h. Then, the withered tea shoots were transferred into a rotary machine (6CWL-90, Fujian Jiayou Machine Co., Ltd., Quanzhou, China) for shaking. The first shaking lasted 4 min at a speed of 30 revolutions per min (r/min), followed by a standing time of 1.5 h. The second shaking lasted 6 min, with a standing time of 2.5 h. The third shaking lasted 10 min, with a standing time of 4 h. After the shaking process, all leaves were transferred into a drum roasting machine and roasted at 290 °C for 2.5 min. Then, the leaves were rolled while hot without pressure for 1 min and under medium-pressure rolling for 5 min. The drying process was initially performed at 120 °C for 30 min, and then at 90 °C for 2 h by using a hot-air drying apparatus (DHG-9123A, Shanghai Qixin Machine Co., Ltd., Shanghai, China). In the different processing processes, except for a single process design parameter, other processes were completed according to the above determined parameters.

### 2.3. Single-Factor Experiment

A single-factor experiment for the withering step: a batch of freshly picked tea shoots was divided into 5 equal parts and placed 15 cm away from the lamp. Each part was 3 cm thick and surrounded by black abrasive cloth. They were respectively equipped with red light (RL), yellow light (YL), and blue light (BL) LED tubes. The light intensity was measured at 492 Lux, with the red light having a wavelength of 680 nm, yellow light having a wavelength of 580 nm, and blue light having a wavelength of 450 nm (Shenzhen Tieniu Information Technology Co., Ltd., Shenzhen, China). The control group used white light (WL) for withering. Samples were exposed at intervals of 0, 2, 4, 6, 8, 12,16, 20, and 25 h [2,22,23]. Each sample consisted of three biological replicates.

A single-factor experiment for the leaf rotating step: the withered leaves were divided into five parts for rotating as presented in Table 1 [7,8]. Among them, A2 served as the control group. After sampling was completed, each sample consisted of three biological replicates.

A single-factor experiment for the fixing step: after withering and leaf rotating, the leaves were divided into 15 parts, and 15 different processes were used for fixing as presented in Table 2 [21,24,25]. F3 served as the control group. At the end of each process, three biological replicates were taken as samples.

A single-factor experiment for the rolling step: the leaves were divided into four parts after withering with white light, leaf rotating, and fixing as presented in Table 3, and then subjected to hot rolling (HR), hot-room temperature rolling (H-RTR), room-temperature rolling, and low-temperature rolling (LTR), respectively [26,27]. HR served as the control group. Samples were collected at the end of each rolling process, with three biological replicates for each sample.

A single-factor experiment for the drying step: the rolled leaves were divided into four parts after withering with white light, leaf rotating, fixing, and rolling as presented in Table 4 [14,28,29]. They were then dried using hot-air drying (HD), pan-fire drying (PD), microwave drying (MD), and freeze drying (FD) (Christ Delta 1-24 LSC, Martin Christ, Osterode, Germany) for drying operations. Samples were taken after drying, and each sample was composed of three biological replicates.

### 2.4. Effects of Different Comprehensive Processing Techniques on the Retention of Catechins

**Figure 1:**
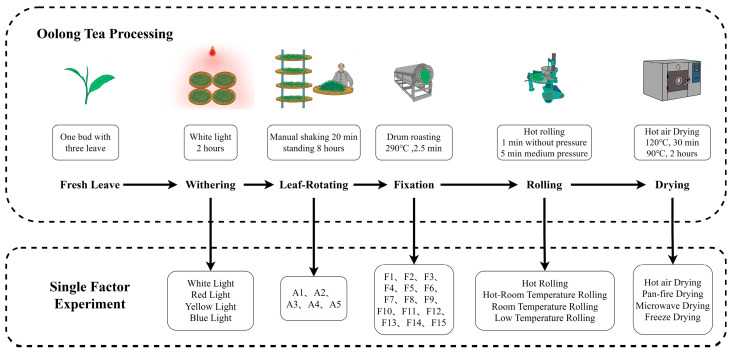
The oolong tea processing and single-factor experiment.

According to the results of a single-factor experiment, an orthogonal experiment with four factors and three levels was conducted. The four factors were the withering process (A), fixing process (B), rolling process (C), and drying process (D). The control group consisted of oolong tea processing technology (Figure 1). Samples were taken after processing, and each sample included three biological repeats.

### 2.5. Detection of Catechin Content

The content of catechins was determined according to the protocol reported in a previous study [30]. Briefly, an ultra-high performance liquid chromatograph (Waters ACQUITY UPLC H-Class, Waters ACQUITY UPLC HSST3 column) was used to detect the contents of GA, EGC, C, EC, EGCG, ECG, GCG, and CG. Chromatographic conditions were conducted as follows: flow rate (0.4 mL/min), column oven temperature (35 °C). Representative wavelengths were at 278 nm for catechins. Mobile phase A: 90 mL acetonitrile, 20 mL acetic acid, 2 mL EDTA, and 888 mL water, shake and then filter the solution with a 0.45 μM membrane. Mobile phase B: 800 mL acetonitrile, 20 mL acetic acid, 2 mL EDTA, and 178 mL water, and then filter the solution with a 0.45 μM membrane. During sample detection, conduct a blank solvent analysis every 10 samples and verify system stability through chromatographic comparison.

Samples for UPLC prepared: Plant tissue was uniformly ground and then extracted with 70% methanol solution in a water bath at 70 °C. Sample (0.2 g (accurate to 0.0001 g)) was placed in 10 mL centrifuge tube, and 5 mL of preheated (70 °C) 70% methanol solution was added. The mixture was stirred with a glass rod and placed into a 70 °C water bath. Extraction was performed after 10 min (stirring once every 5 min) and after cooling to room temperature, transferred to centrifuge, and spun at 3500 r/min centrifugal for 10 min. The supernatant was then transferred to a 10 mL volumetric flask. Residue and then 70% methanol solution 5 mL extracted once, and repeated as above. The combined extract volume was made up to 10 mL, shaken, then the solution was filtered with a 0.45 μM membrane, and stored at 4 °C for up to 24 h before being analyzed by UPLC.

### 2.6. Statistical Analysis

Each sample consists of three biological replicates. All experimental data were calculated as the average of three replicate experiments and reported as the means ± standard deviations. SPSS (Version 25, SPSS Inc., Chicago, IL, USA) was used to perform principal component analysis (PCA) and analyze the significant differences among different treatments. Duncan’s test was used to analyze the significance of differences. Origin software (version 2021, Origin Lab Corp., Northampton, MA, USA) was used for correlation analysis. SIMCA 14.1 software (Umetrics, Umea, Sweden) was used to perform orthogonal partial least square discriminant analysis (OPLS-DA) and variable importance in projection (VIP) analysis. Retention rate = component content in samples from different processes/component content in fresh leaves. Catechin bitterness index = (EGCG + EGC + ECG + GC)/(C + EC) [31,32]. Total simple catechins (TSC) = EGC + EC + C. Total ester-type catechins (TETC) = EGCG + GCG + ECG + CG. 

### 2.7. Sensory Evaluation

The sensory evaluation panel consisted of eleven members professionally engaged in tea production and organoleptic assessment. The members of the panel comprised 6 males and 5 females ranging from 30 to 50 years old. The preparing procedures of tea infusions were conducted according to the Chinese national standard (GB/T 23776-2018) [33]: 110 mL of boiling water was added to 5 g of each tea sample in separate teacups with their lids for 5 min to obtain a tea infusion. Then, the score (0–100), taste descriptors (mellow, bitterness, umami, astringency, and thick), and aroma descriptors (pure, floral, fruity, grassy, and dull) of each tea sample infusion were subjected to a sensory test by the eleven panelists [10,34]. All panelists of the sensory evaluation panel were informed and the informed consent was obtained from all participants for our experiments.

## 3. Results

### 3.1. Changes of Catechins in Different Withering Methods

**Figure 2:**
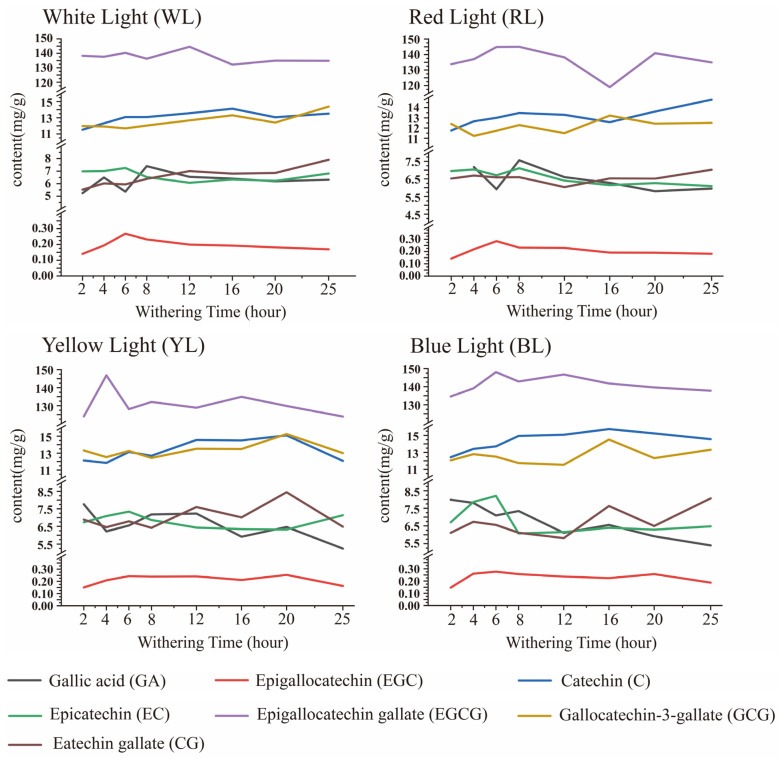
Catechin component content after withering of different light quality, RL: red light, WL: white light, YL: yellow light, BL: blue light.

After being treated with different light qualities and varying withering times, the trend of different catechin compounds during withering under WL, YL, RL, and BL exhibited a wave-like pattern (Figure 2). The peak value of TC was 12 h in WL (Table S2), 4 h in YL (Table S3), 8 h in RL (Table S4), and 6 h in BL (Table S5). 

**Figure 3:**
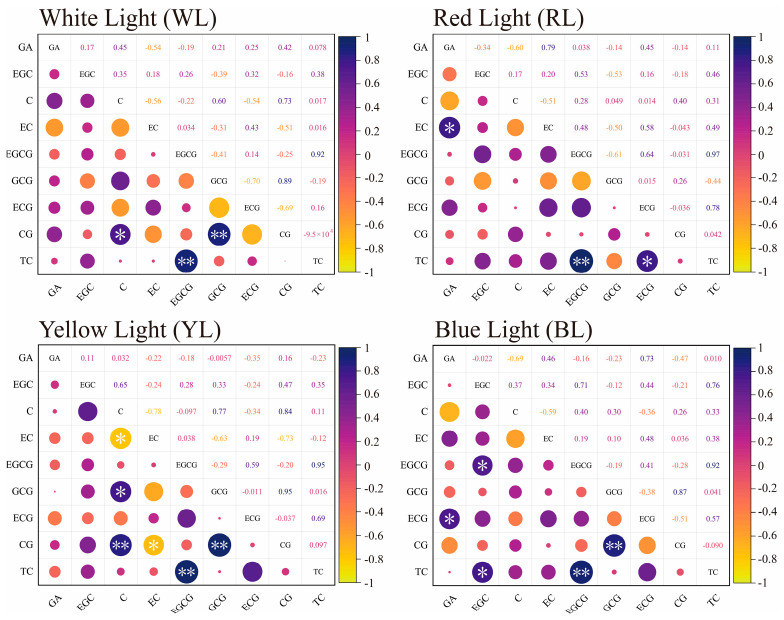
Correlation of catechin components of different light quality withering, RL: red light, WL: white light, YL: yellow light, BL: blue light, ** indicates *p* < 0.01, * indicates *p* < 0.05, ns indicates *p* > 0.05.

Under the four withering methods, the EGCG content decreased rapidly during the first two hours before withering and then gradually decreased as the withering time extended (Tables S1–S4). The peak value of EGCG was 12 h in WL (Table S2), 4 h in YL (Table S3), and 8 h in RL (Table S4). The trend of EGCG was similar to the change observed for the TC of four withering methods. Correlation analysis revealed a positive correlation (r > 0.6) between EGCG and TC across all four light quality treatments (Figure 3).

During the later stage of withering, the levels of EGC, EC, ECG, and GA gradually declined after reaching their respective peak values, while the concentrations of CG, C, and GCG exhibited a continuous increase (Figure 2). Compared to fresh leaves, the CG exhibited a 37% increase under WL 25 h (Table S2), a 46% increase under YL 20 h (Table S3), a 21% increase under RL 25 h (Table S4), and a 39% increase under BL 25 h (Table S5). C increased by 32% in WL 16 h (Table S2), increased by 41% in YL 20 h (Table S3), increased by 38% in RL 25 h (Table S4), and increased by 48% in BL 16 h (Table S5). GCG increased by 13% in WL 25 h (Table S2), increased by 20% in YL 20 h (Table S3), increased by 4% in RL 16 h (Table S4), and increased by 14% in BL 16 h (Table S5).

The correlation between the content of C, CG, GCG, EC, ECG, and EGC under different light qualities was analyzed (Figure 3). A significant negative correlation was observed between EC and C, EC and CG in YL treatment. Moreover, dramatically positive correlations were found between C and GCG, C and EGC, and C and CG; similarly, CG exhibited a significant positive correlation with GCG (*p* < 0.05). A negative correlation is observed between EC and C, as well as between EGC and GCG in RL treatment; additionally, a significant positive correlation exists between C and CG. Conversely, a negative correlation is found between ECG and CG, while CG and GCG exhibit a significantly positive correlation in BL treatment. 

**Figure 4:**
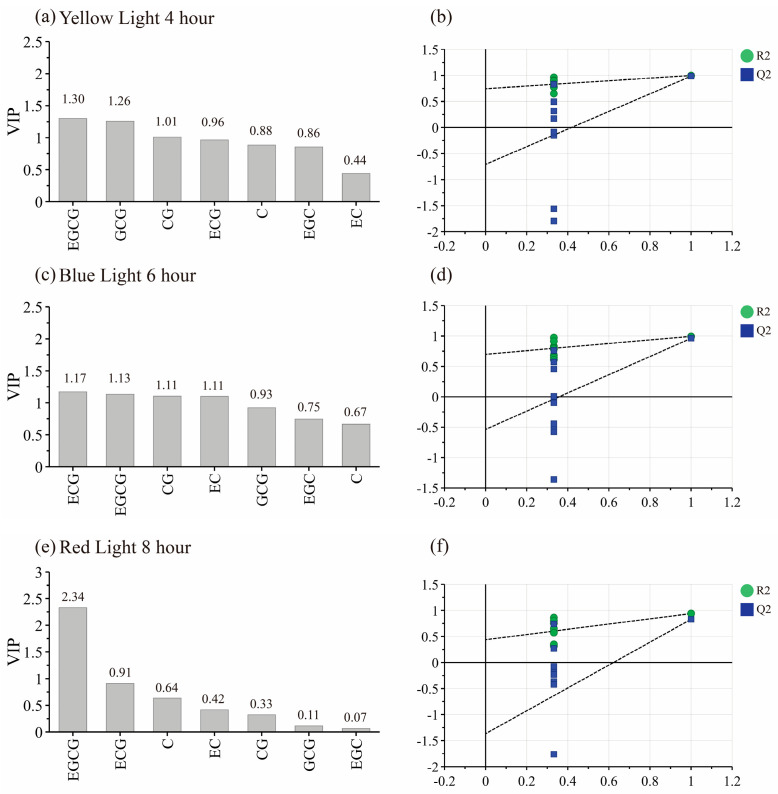
VIP plot and cross-validation plot of the OPLS-DA model, (**a**) VIP plot of yellow light withering 4 h, (**b**) cross-validation plot of the OPLS-DA model, R2 = 0.705, Q2 = 0.985, (**c**) VIP plot of blue light withering 6 h, (**d**) cross-validation plot of the OPLS-DA model, R2 = 0.836, Q2 = 0.962, (**e**) VIP plot of red light withering 8 h, (**f**) cross-validation plot of the OPLS-DA model, R2 = 0.924, Q2 = 0.83.

The levels of TC were compared (Tables S2–S5) and a significant preservation of TC was observed after YL 4 h, BL 6 h, and RL 8 h. OPLS-DA models (Figure 4) were established for YL 4 h, BL 6 h, RL 8 h and WL 4 h, WL 6 h, and WL 8 h, respectively, according to the principle of a VIP value over one. Compared with WL 4 h, YL 4 h mainly affected EGCG, GCG, and CG. Compared with WL 6 h, BL 6 h mainly affected EGC, EGCG, EC, and CG. Compared with WL 8 h, RL 8 h mainly affected EGCG. The retention of more EGCG may potentially lead to an increase in TC.

### 3.2. Changes of Catechins in Different Leaf Rotating Methods 

**Figure 5:**
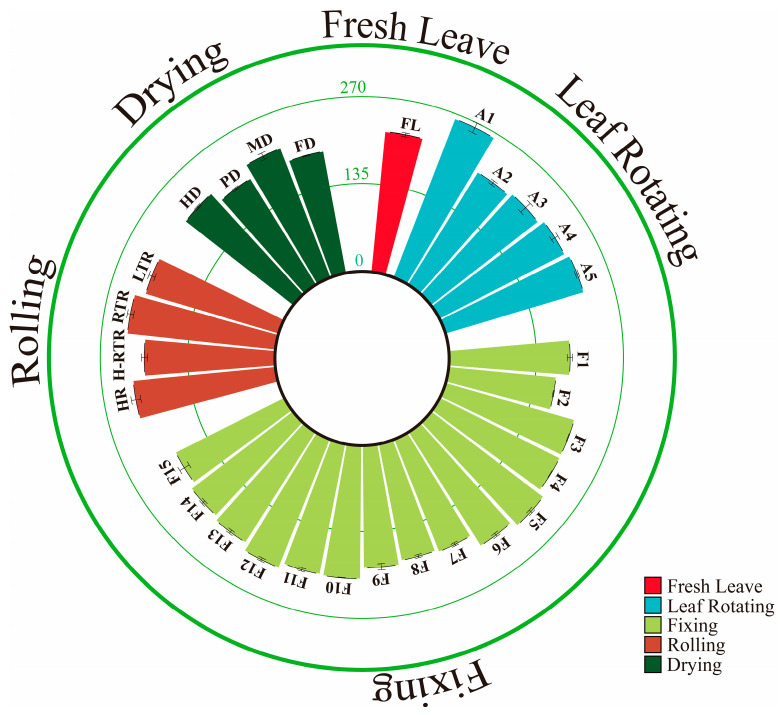
Total catechin content in different processing processes. FL: fresh leaf; A1, A2, A3, A4, A5 represent different leaf rotating methods; F1, F2, F3, F4, F5, F6, F7, F8, F9, F10, F11, F12, F13, F14, F15 represent different fixation methods. HR: hot rolling, H-RTR: hot-room-temperature rolling, RTR: room-temperature rolling, LTR: low-temperature rolling; HD: hot-air drying, PD: pan-firing drying, MD: microwave drying, FD: freeze drying.

The retention rates of EGCG, TSC, TETC, and TC were A1 > A5 > A4 (Figure 5). Compared with A2 (Table 5), EGCG in the group A1 was significantly increased by 43.18% (*p* < 0.01), TSC was significantly increased by 41.21% (*p* < 0.01), TETC was significantly increased by 25.26% (*p* < 0.01), and TC was significantly increased by 27.70% (*p* < 0.01). The TETC/TSC ratio and bitter astringency index of A1 were both low (Table 5), suggesting that A1 exhibits a propensity for a high catechin content and a low bitter astringency in oolong tea production.

### 3.3. Changes of Catechins in Different Fixing Methods 

At the end of fixing, the TSC in F6 (Table S6) increased significantly by 45.97% (*p* < 0.05), and the TETC in F5 and F12 increased significantly by 4.96% and 3.38% (*p* < 0.05). The TC in F5 increased significantly by 5.57% (*p* < 0.05). The TC obtained from microwave and steam treatment exhibited higher levels compared to those acquired through pan-firing and drum-roasting treatment. Compared with TC, TETC, and EGCG, the retention effect of F5 exhibited a better performance. 

### 3.4. Changes of Catechins under Different Rolling Methods 

At the end of rolling, the TC of RTR (Table 6) was the highest. For RTR compared with HR, TC of RTR increased by 4.3% (*p* < 0.05), TSC increased by 6.0% (*p* < 0.05), TETC increased by 3.9% (*p* < 0.05), and EGCG increased by 3.5%. The TC of H-RTR was the lowest. For H-RTR compared with HR, TC decreased by 8.5% (*p* < 0.05), TSC decreased by 7.8% (*p* < 0.05), TETC decreased by 11.7% (*p* < 0.05), and the EGCG content decreased by 7.5% (*p* < 0.05). Comprehensive comparison of EGCG, TSC, TETC, and TC retention rates showed RTR > HR > H-RTR > LTR. 

### 3.5. Changes of Catechins under Different Drying Methods

After MD (Table 7), TC reached the maximum. For MD compared with HD, the TC increased by 1.6%, TETC increased by 3.9% (*p* < 0.05), TSC decreased by 9.9% (*p* < 0.05), and EGCG increased by 0.2%. After FD, the retention rate of TC was the lowest. Compared with the HD, TC decreased by 9.1% (*p* < 0.05), TETC decreased by 11% (*p* < 0.05), TSC increased by 0.6%, and EGCG content decreased by 11.7% (*p* < 0.05). After PD, TSC decreased by 11.3% (*p* < 0.05). Overall, higher retention rates for EGCG, TETC, and TC were found in MD.

### 3.6. Changes of Catechins under the Combination of Comprehensive Processing Methods

Based on the results of single-factor experiments on withering, leaf rotating, fixing, rolling, and drying, an orthogonal combination experiment scheme was designed with four factors and three levels (Table 8 and Table 9). Compared to CK (Table S7), significant increases in TC were observed in OE3, OE4, OE8, OE9, OE7, and OE5 (*p* < 0.05), with respective increments of 23.7%, 16.8%, 11.6%, 6.7%, 4.6%, and 2.7%. The retention content of TETC in OE3, OE4, OE5, OE7, and OE9 was significantly increased compared to CK (*p* < 0.05). The retention content of TSC in both OE3 and OE4 was significantly increased compared to CK (*p* < 0.05). Except for OE2, EGCG levels were significantly higher than CK in all other groups (*p* < 0.05). Overall, OE3 showed higher retention rates for EGCG, TETC, and TC.

Principal component analysis was carried out on nine different orthogonal combinations, and the function expressions of Principal Components 1, 2, and 3 of the different processing techniques were obtained as follows:

F1 = 0.187 ∗ X1 − 0.239 ∗ X2 + 0.491 ∗ X3 + 0.453 ∗ X4 + 0.502 ∗ X5 + 0.404 ∗ X6 + 0.214 ∗ X7

F2 = −0.001 ∗ X1 + 0.538 ∗ X2 + 0.195 ∗ X3 − 0.037 ∗ X4 − 0.016 ∗ X5 + 0.491 ∗ X6 − 0.655 ∗ X7

F3 = −0.797 ∗ X1 + 0.313 ∗ X2 − 0.058 ∗ X3 − 0.076 ∗ X4 + 0.236 ∗ X5 + 0.209 ∗ X6 + 0.396 ∗ X7

In the formula, F1, F2, and F3 represent the weight values of the feature vectors corresponding to the first, second, and third principal components of different processing techniques, respectively. X1, X2, X3, X4, X5, X6, and X7 represent the standardized results of EGC, C, EC, EGCG, GCG, ECG, and CG. The processing techniques were ranked based on their comprehensive scores as follows: OE3 > OE4 > OE8 > CK > OE5 > OE1 > OE7 > OE9 > OE6 > OE2. 

The catechin components of different orthogonal combinations were analyzed through correlation analysis. After OE2 treatment (Figure S1), TC showed significant positive correlations with EGCG, GCG, and ECG, while showing negative correlations with GA, EGC, and CG. After OE3 treatment (Figure S2), TC exhibited positive correlations with all other components except C. Among the nine processing methods compared for the retention of EGCG, GCG, ECG, and CG, OE3 performed the best. OPLS-DA analysis was conducted on OE3, OE4, and OE8 (Figure S3), according to the principle of a VIP value over 1.0, and it was found that OE3 mainly regulates EGCG and GCG, while OE4 mainly regulates EGCG, and OE8 mainly regulates both EGCG and GCG. These findings suggest that effective processing techniques require the regulation of EGCG.

### 3.7. Sensory Evaluation of Oolong Tea under the Combination of Comprehensive Processing Methods

The sensory quality of comprehensive processing methods was investigated. The sensory evaluation results are shown in Table 10. Compared to CK, OE4 has a fresh and lasting aroma, the taste is mellow and brisk, with typical quality characteristics of oolong tea. OE3 has the aroma of fruit, the taste is mellow and lightly bitter. OE2 has the aroma of caramel and the taste is mellow and grassy. The scores for sensory evaluation are as follows: OE4 > OE3 > CK > OE5 > OE9 > OE2 > OE1 > OE8 > OE6 > OE7. Oolong tea with a high catechin content is more mellow and brisk in taste, and the tea infusion is brighter.

## 4. Discussion

### 4.1. Degradation of EGCG Led to the Change in Catechin Components in the Enzymatic Oxidation Stage

In the withering single-factor experiment, according to the principle of a VIP value over one, we can find that EGCG is the main regulated component in four different light qualities (Figure 4). The accumulation of EGC, EC, ECG, and GA during the withering may be attributed to the lower redox potential of the trihydroxybenzene ring of EGCG [35]; the increased levels of EGC and GA (Tables S2–S5) are due to the conversion of EGCG. Correlation analysis in this study showed that the accumulation of C, CG, and GCG was related to EC, EGC, and GA (Figure 3). The same trend in C, CG, and GCG levels may be due to the synergistic effect of the environment and leaf enzyme activity [36,37]. The leaves undergo continuous water loss and exhibit obvious shrinkage of vacuoles with the increase in withering degree [38]. Consequently, there is a significant down-regulation in the activities of phenylalanine ammonia lyase and chalcone synthetase [17], which may lead to a decrease in EGC, EC, ECG, and GA during the later stages of withering (Figure 2). The isomerization of EGC, EC, ECG, and GA by polyphenol oxidase and other hydrolases resulted in the conversion of cis-catechins to trans-catechins, ultimately leading to the accumulation of CG, C, and GCG during the withering stage (Tables S2–S5). The delayed accumulation of GCG exhibited a similar pattern to the significant increase in GCG content observed after 9 h of red light withering during black tea processing [39]. 

**Figure 6:**
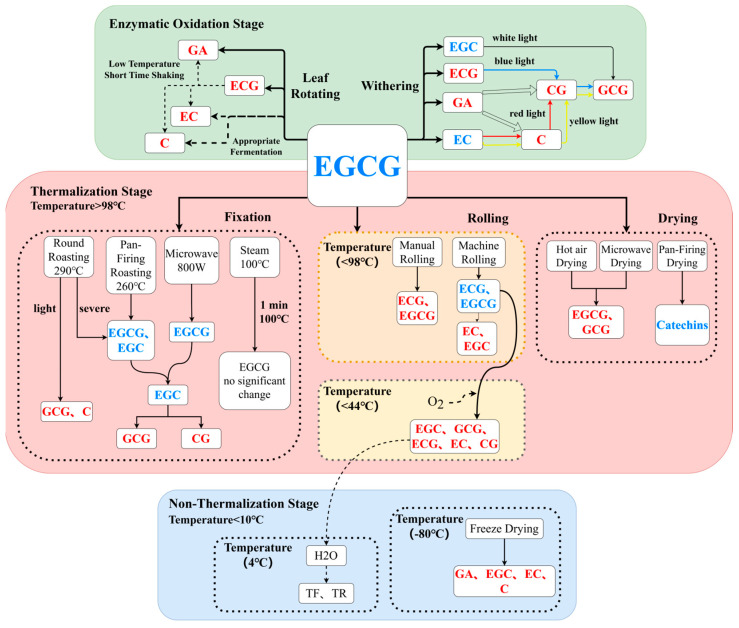
The changes in EGCG in different processing stages. The red font is up-regulated and the blue font is down-regulated. The dotted line is represented as a possible path.

During the leaf rotating single-factor experiment, leaves undergo two steps, including shaking and standing. The time of leaf rotating was within 8 h, which EGCG degraded into GA, ECG, EC, and C through continuous mechanical damage and standing, resulting in a lower bitterness index (Table 5). With the increase in shaking time, the TC (Table 5) decreased significantly (*p* < 0.05). This is consistent with the result that lightly shaking tea produced a higher total flavonoid content than heavily shaking tea [5]. Compared with the A2 (Table 5) and fresh leaves (Table S1), A1 exhibited a significant increase in TC (*p* < 0.05), indicating that a moderate shaking intensity and standing time could enhance the accumulation of TC (Figure 6). The general oolong tea fermentation environment temperature is low. The activity of polyphenol oxidase (PPO) was higher at lower temperatures (20 °C and 25 °C) [40]. Shaking and standing for a short time reduce the leaf temperature and increase the activity of polyphenol oxidase, which catalyzes the decomposition of ester catechins such as EGCG and EGC into non-ester catechins and gallic acid [41]. This may be the reason why A1 (Table 5) has a higher EC content compared to other treatments. The increase in the standing time leads to a decrease in the retention rate of EGCG in leaves (Table 5), but the TC increases when the standing time is controlled within 16 h [42]. This may be attributed to the mechanical stress caused by the collision of fresh tea leaves during shaking. The permeability of cell membranes increases under conditions of stress and cell damage, which enhances the possibility of contact between enzymes and substrates, thereby promoting the oxidative decomposition of catechins. The peroxidase activity in black tea fermentation is directly proportional to the temperature, whereby higher temperatures accelerate the rate of oxidation depletion of catechins [43]. The prolonged duration of standing leads to an elevation in the internal temperature of the leaves, which hinders the preservation of catechins. Optimal fermentation time (Figure 6) facilitates the oxidation, hydrolysis, polymerization, and transformation of ester catechin into dimeric catechins while significantly enhancing the content of non-ester catechins [44]. It provides a solid basis for further material transformation.

### 4.2. Thermalization Affects Catechin Components in the Non-Enzymatic Stage

Under heavy baking, most of the catechins in green tea were degraded [45]. The isomerization, hydrolysis, and oxidation of catechin components typically occur subsequent to exposure to high temperatures [46]. In particular, epicatechins are prone to isomerization reactions under high-temperature conditions [47,48]. The changes in EGCG and EGC to CG and GCG occur during the fixation of green tea [27]. In the fixing single-factor experiment, the TETC decreased significantly after four different fixation methods (Table S6), and EGCG decreased significantly (*p* < 0.05). The levels of EGC, EC, ECG, and EGCG exhibited a gradual decline over time during both microwave fixation and steam fixation. Under mild drum baking, the proportion of non-epi-catechins in TC increased, and the proportion of epicatechins decreased [45]. Compared with F1 and F3 (Table S6), mild drum roasting in F1 had higher non-epicatechins (GCG, C) and lower epicatechins (EGCG, EGC) than drum fixation in F3. When the temperature exceeds 98 °C, EGCG primarily undergoes isomerization [49]. The temperatures of microwave fixation F4, F5, F6, F7, F8, and F9 (Table 2) and steam fixation F10, F11, F12, F13, F14, and F15 (Table 2) all exceeded 98 °C. The lack of significant degradation or isomerization of EGCG being observed during the process may be due to the brief exposure to a high temperature. When the temperature reached 165 °C, EGCG decreased by approximately 50% in 5 min. The isomerization and degradation of EGCG exhibited conformity with first-order reaction kinetics. Furthermore, both the degradation and isomerization reactions were influenced by variations in temperature [50]. Similar results were also obtained in this study: the EGCG content was lower than other treatments at 260 °C pan-firing fixing (Table S6).

The traditional hand-rolling method employed in the production of Yunnan black tea and raw tea allows for a higher retention of ECG and EGCG [51]. However, the content of ester catechins such as EGCG and GCG was found to be low when oolong tea was mechanically rolled while hot [26]. Similar results were obtained in this study following hot rolling. The disparity in EGCG content retention between hand-rolling and machine-rolling may be attributed to variances in applied rolling pressures. The use of a rolling machine for rolling can effectively curl the leaves, thereby accelerating the oxidative degradation of catechins. Simultaneously, the thermal effect can further enhance the hydrolysis of total ester-type catechins, leading to the conversion of ECG and EGCG into EC and EGC [52,53]. In the rolling single-factor experiment (Table 6), the highest catechin content was obtained under RTR, which may be related to the chemical structure of EGCG. At the temperature of 44 °C, EGCG is mainly degraded, while GCG is difficult to isomerize to EGCG [49]. As a result, after being rolled at room temperature, EGCG converts into non-gallated catechins and gallic acid, leading to higher retention rates observed for EGC, GCG, ECG, EC, and CG compared to other treatments (Table 6). The content of EGCG is reduced due to oxidation during the long rolling phase [54]. The content of tea polyphenols in oolong tea showed a downward trend after repeated rolling [55]. The retention rate of TC was the lowest after H-RTR (Table 6). It may be that during the spreading process, catechins are fully exposed to oxygen (Figure 6), leading to condensation polymerization under high temperature and humidity conditions and resulting in a decrease in content [56]. Low temperature can reduce the isomerization of catechins [46]. The LTR has a lower temperature than the RTR, but it does not result in a better retention of various catechin components (Table 6). Previous studies have also found a significant decrease in oolong tea polyphenol content during low-temperature rolling [56]. Similar results were obtained under LTR treatment in this study. During the gradual heating of rolled leaves from a low temperature (4 °C) to room temperature (28 °C), it is possible that condensation occurs, leading to the dissolution and subsequent oxidation of certain catechins into theaflavins and other compounds. Consequently, this phenomenon contributes to a diminished retention rate of total catechins (Table 6).

In the drying single-factor experiment (Table 7), the content of EGCG and GCG in MD was higher than that in other three drying methods, and the retention of TC was more effective under MD. These findings are consistent with the results of green tea drying at different temperatures [29], which may be attributed to the efficient heat transfer performance of microwaves. After the evaporation of leaf water during microwave drying, the leaves cease to generate heat, and a shorter heating time can further prevent oxidation and isomerization of catechins [28]. The content of flavonoids in citrus peel dried by hot air was relatively high [57]. The levels of polyphenols and various catechins in hot-air-dried green brick tea were higher than those in sun-dried green brick tea [58]. In the drying single-factor experiment (Table 7), the TC under HD has a higher retention, and has a lower TETC/TSC value and bitterness index, indicating that hot-air drying can take into account higher catechins’ retention and tea quality. In the experiment, hot-air drying resulted in higher catechins retention and improved tea quality, as indicated by the higher TC under HD, lower TETC/TSC value, and reduced bitterness index. The content of TC was lower in HD compared to MD (Table 7), possibly due to the slower decrease in heat conduction rate and leaf surface temperature, which leads to a prolonged effective heat treatment time and results in a decrease in certain catechins under heat action [28,50]. Freeze-dried broad beans had higher GA and polyphenol retention [59]. Freeze-dried ginkgo biloba leaf tea had higher GA and EGC retention [60]. The lower levels of EGC, EC, and C under HD, PD, and MD compared to FD (Table 7) may be attributed to the susceptibility of EC and EGC to high temperatures and their increased stability at low temperatures [46]. The low retention rate of TSC under PD may be attributed to the degradation of catechin components caused by high-temperature rolling.

## 5. Conclusions

The optimal production procedure of oolong tea balancing high catechin and sensory quality is as follows: red light withering for 8 h, leaf rotating for 10 min with a total standing time for 8 h, drum roasting for 5 min at 290 °C, low-temperature rolling (flattening at 4 °C for 5 min, without pressure for 1 min and under pressure for 5 min), and microwave drying (800 W for 7.5 min). The significant increased retention in catechins contributed to the mellow and brisk tastes of oolong tea, promoting the formation of the characteristic flavor of oolong tea. The results described in this work confirm that the optimal procedures for high catechins’ contents is more conducive to the formation of the characteristic quality of oolong tea than the traditional producing procedures. Our study provides a novel insight into the relationship between the oolong tea processing and flavor formation; however, further investigation on the possible mechanism underlying the conversion or accumulation of the overall profiles of flavor compounds over the entire production process should be conducted to explore the ultimate contribution of the optimal procedure to the final flavor quality of oolong teas.

## Figures and Tables

**Table 1:** Leaf rotating single-factor experiment.

No.	Shaking	Standing
A1	Manual shaking, 30 r/min, 10 min for total shaking time: the first shaking for 2 min; the second shaking for 3 min; the third shaking for 5 min.	8 h for total standing time: the first standing for 1.5 h; the second standing for 2.5 h; the third standing for 4 h.
A2	Manual shaking, 30 r/min, 20 min for total shaking time: the first shaking for 4 min; the second shaking for 6 min; the third shaking for 10 min.	8 h for total standing time: the first standing for 1.5 h; the second standing for 2.5 h; the third standing for 4 h.
A3	Manual shaking, 30 r/min, 30 min for total shaking time: the first shaking for 6 min; the second shaking for 10 min; the third shaking for 14 min.	8 h for total standing time: the first standing for 1.5 h; the second standing for 2.5 h; the third standing for 4 h.
A4	Manual shaking, 30 r/min, 20 min for total shaking time: the first shaking for 4 min; the second shaking for 6 min; the third shaking for 10 min.	6 h for total standing time: the first standing for 1 h; the second standing for 2 h; the third standing for 3 h.
A5	Manual shaking, 30 r/min, 20 min for total shaking time: the first shaking for 4 min; the second shaking for 6 min; the third shaking for 10 min.	10 h for total standing time: the first standing for 2 h; the second standing for 3 h; the third standing for 5 h.

**Table 2:** Fixing single-factor experiment.

No.	Fixings
F1	Drum roasting at 290 °C for 2 min, microwave at 800 W for 30 s
F2	Pan-firing roasting at 260 °C for 4 min
F3	Drum roasting at 290 °C for 2.5 min
F4	Microwave at 800 W for 10 s
F5	Microwave at 800 W for 20 s
F6	Microwave at 800 W for 30 s
F7	Microwave at 800 W for 40 s
F8	Microwave at 800 W for 50 s
F9	Microwave at 800 W for 60 s
F10	Steam at 100 °C for 10 s
F11	Steam at 100 °C for 20 s
F12	Steam at 100 °C for 30 s
F13	Steam at 100 °C for 40 s
F14	Steam at 100 °C for 50 s
F15	Steam at 100 °C for 60 s

**Table 3:** Rolling single-factor experiment.

No.	Rolling
HR	Rolling while hot, no pressure rolling for 1 min, middle pressure rolling for 5 min
H-RTR	Rolling while hot, no pressure rolling for 1 min, spreading for 5 min, middle pressure rolling for 5 min
RTR	Spreading for 5 min, no pressure rolling for 1 min, middle pressure rolling for 5 min
LTR	Spreading at 4 °C for 5 min, no pressure rolling for 1 min, middle pressure rolling for 5 min

**Table 4:** Drying single-factor experiment.

No.	Drying
HD	Hot-air baking at 120 °C for 30 min, 90 °C for 120 min
PD	Pan-firing for 90 min
MD	Microwave at 800 W for 8 min
FD	Freeze drying for 4 h

**Table 5:** Catechins’ contents of different leaf rotating methods.

Compound	Treatment				
	A1	A2	A3	A4	A5
Gallic acid	12.15 ± 0.64 a	10.96 ± 0.31 b	10.98 ± 0.32 b	10.35 ± 0.26 b	10.62 ± 0.31 b
Epigallocatechin	0.28 ± 0.07 b	0.09 ± 0.00 c	0.42 ± 0.03 a	0.25 ± 0.06 b	0.42 ± 0.01 a
Catechin	12.30 ± 0.44 a	9.95 ± 0.17 b	9.94 ± 0.77 b	10.64 ± 0.04 b	10.30 ± 0.26 b
Epicatechin	31.25 ± 1.01 a	21.47 ± 1.24 c	22.42 ± 1.23 bc	21.26 ± 1.39 c	24.26 ± 0.48 b
Epigallocatechin gallate	147.83 ± 3.74 a	102.95 ± 2.04 d	109.46 ± 4.29 c	118.63 ± 3.52 b	123.38 ± 2.08 b
Gallocatechin-3-gallate	20.75 ± 0.96 a	19.06 ± 0.67 b	19.79 ± 0.27 ab	21.11 ± 1.14 a	20.42 ± 0.25 ab
Epicatechin-3-gallate	31.19 ± 1.46 b	33.83 ± 0.52 a	28.76 ± 2.55 c	28.44 ± 0.87 c	27.40 ± 0.36 c
Catechin gallate	13.59 ± 0.48 ab	12.77 ± 0.30 bc	14.33 ± 0.59 a	12.13 ± 0.64 cd	11.63 ± 0.39 d
Total simple catechin	43.83 ± 1.35 a	31.51 ± 1.27 c	32.77 ± 2.02 bc	32.16 ± 1.34 c	34.98 ± 0.43 b
Total ester-type catechin	213.35 ± 6.60 a	168.61 ± 2.19 d	172.34 ± 7.67 cd	180.32 ± 4.46 bc	182.83 ± 2.30 b
TETC/TSC	4.87 ± 0.03 c	5.35 ± 0.15 b	5.26 ± 0.09 b	5.61 ± 0.15 a	5.23 ± 0.08 b
Total catechin	257.18 ± 7.92 a	200.12 ± 3.46 d	205.11 ± 9.68 cd	212.48 ± 5.59 bc	217.81 ± 2.42 b
Bitterness index	4.43 ± 0.04 c	4.77 ± 0.10 b	4.73 ± 0.06 b	5.00 ± 0.14 a	4.71 ± 0.08 b

TSC: total simple catechin, TETC: total ester-type catechin. Values in the same row followed by different lowercase letters are significantly different at *p* < 0.05.

**Table 6:** Catechins’ contents of different rolling methods.

Compound	Treatment			
	HR	H-RTR	RTR	LTR
Gallic acid	10.23 ± 0.56 b	10.39 ± 0.21 b	11.61 ± 0.56 a	10.63 ± 0.17 b
Epigallocatechin	0.13 ± 0.01 b	0.11 ± 0.01 b	0.22 ± 0.02 a	0.11 ± 0.01 b
Catechin	11.07 ± 0.42 a	9.91 ± 0.37 c	10.11 ± 0.09 bc	10.54 ± 0.17 ab
Epicatechin	22.27 ± 0.87 b	19.52 ± 0.53 c	25.16 ± 0.51 a	21.21 ± 0.64 b
Epigallocatechin gallate	123.44 ± 4.97 a	114.24 ± 2.25 b	127.79 ± 1.85 a	122.24 ± 3.33 a
Gallocatechin-3-gallate	19.37 ± 0.45 b	17.42 ± 0.90 c	20.83 ± 0.82 a	17.82 ± 0.59 c
Epicatechin-3-gallate	34.53 ± 0.59 a	31.56 ± 1.56 b	35.13 ± 1.60 a	33.11 ± 0.44 ab
Catechin gallate	4.34 ± 0.28 b	4.21 ± 0.21 b	5.08 ± 0.19 a	4.23 ± 0.06 b
Total simple catechin	33.47 ± 1.16 b	29.54 ± 0.85 c	35.49 ± 0.43 a	31.86 ± 0.81 d
Total ester-type catechin	181.68 ± 6.24 ab	167.42 ± 4.38 c	188.84 ± 4.35 a	177.41 ± 4.21 b
TETC/TSC	5.43 ± 0.14 ab	5.67 ± 0.19 a	5.32 ± 0.06 b	5.57 ± 0.08 ab
Total catechin	215.15 ± 7.11 ab	196.97 ± 4.66 c	224.34 ± 4.77 a	209.27 ± 4.90 b
Bitterness index	4.87 ± 0.13 bc	5.10 ± 0.17 a	4.77 ± 0.05 c	5.03 ± 0.08 ab

TSC: total simple catechin, TETC: total ester-type catechin. Values in the same row followed by different lowercase letters are significantly different at *p* < 0.05.

**Table 7:** Catechins’ contents under different drying methods.

Compound	Treatment			
	HD	PD	MD	FD
Gallic acid	11.26 ± 0.16 a	8.53 ± 0.34 b	10.84 ± 0.11 a	11.16 ± 0.27 a
Epigallocatechin	0.18 ± 0.01 b	0.16 ± 0.03 c	0.22 ± 0.01 b	0.35 ± 0.04 a
Catechin	10.72 ± 0.13 b	10.55 ± 0.22 b	10.85 ± 0.17 b	11.27 ± 0.24 a
Epicatechin	22.84 ± 0.28 a	19.22 ± 0.78 b	19.33 ± 0.58 b	22.33 ± 0.57 a
Epigallocatechin gallate	115.08 ± 0.69 a	109.03 ± 0.48 b	115.36 ± 5.43 a	101.64 ± 0.49 c
Gallocatechin-3-gallate	19.54 ± 0.43 b	17.18 ± 0.20 d	26.17 ± 0.33 a	18.52 ± 0.44 c
Epicatechin-3-gallate	33.11 ± 1.28 a	28.94 ± 0.51 b	32.23 ± 0.51 a	28.66 ± 1.36 b
Catechin gallate	4.83 ± 0.61 ab	3.84 ± 0.27 b	5.43 ± 0.10 a	4.71 ± 0.18 c
Total simple catechin	33.74 ± 0.20 a	29.93 ± 0.75 b	30.40 ± 0.75 b	33.94 ± 0.78 a
Total ester-type catechin	172.55 ± 1.68 b	158.99 ± 1.15 c	179.20 ± 4.66 a	153.52 ± 1.61 d
TETC/TSC	5.11 ± 0.04 b	5.31 ± 0.11 b	5.90 ± 0.10 a	4.53 ± 0.15 c
Total catechin	206.29 ± 1.81 a	188.91 ± 1.75 b	209.60 ± 5.26 a	187.46 ± 0.83 b
Bitterness index	4.57 ± 0.04 c	4.77 ± 0.10 b	5.08 ± 0.11 a	4.03 ± 0.13 d

TSC: total simple catechin, TETC: total ester-type catechin. Values in the same row followed by different lowercase letters are significantly different at *p* < 0.05.

**Table 8:** Optimization orthogonal design.

Level	Factor			
	Withering (A)	Fixing (B)	Rolling (C)	Drying (D)
1	RL8	F5	HR	HD
2	BL6	F12	RTR	PD
3	YL4	F3	LTR	MD

RL8: red light 8 h, BL6: blue light 6 h, YL4: yellow light 4 h; F5: microwave at 800 W for 20 s, F12: steam at 100 °C for 30 s, F3: drum roasting at 290 °C for 2.5 min; HR: hot rolling, RTR: room-temperature rolling, LTR: low-temperature rolling; HD: hot-air drying, PD: pan-fire drying, MD: microwave drying.

**Table 9:** Optimization orthogonal experiment.

No.	Withering (A)	Fixing (B)	Rolling (C)	Drying (D)
OE1	RL8	F5	HR	HD
OE2	RL8	F12	RTR	PD
OE3	RL8	F3	LTR	MD
OE4	BL6	F5	RTR	MD
OE5	BL6	F12	LTR	HD
OE6	BL6	F3	HR	PD
OE7	YL4	F5	LTR	PD
OE8	YL4	F12	HR	MD
OE9	YL4	F3	RTR	HD

RL8: red light 8 h, BL6: blue light 6 h, YL4: yellow light 4 h; F5: microwave at 800 W for 20 s, F12: steam at 100 °C for 30 s, F3: drum roasting at 290 °C for 2.5 min; HR: hot rolling, RTR: room-temperature rolling, LTR: low-temperature rolling; HD: hot-air drying, PD: pan-fire drying, MD: microwave drying.

**Table 10:** Sensory evaluation of oolong tea under the combination of comprehensive processing methods.

NO.	Appearance	Liquor Color	Aroma	Taste	Infused Leaf	Score
CK	Even color and lightly dry	Orange	Pure and sweet	Mellow and lightly astringent	Even and lightly dull	86.27
OE1	Lightly mixed and lightly dry	Lightly dull yellow	Sweet and lightly caramel	Mellow and lightly coarse	Even and light dull	84.68
OE2	Lightly dull dry	Orange red	Sweet and lightly caramel	Mellow with grassy	Even and lightly dark	84.70
OE3	Even color and smooth	Bright yellow	Fruity and pure	Mellow and lightly bitter	Even	88.60
OE4	Smooth and lightly dry	Yellow	Clean and fresh, lasting	Mellow and brisk	Even	89.25
OE5	Even color and clean	Light yellow	Pure and sweet	Mellow and lightly astringent	Even	85.83
OE6	Even color and lightly dry	Yellow	Grassy	Coarse and astringent	Dull and lightly dark	82.23
OE7	Even color	Light yellow	Less pure and grassy	Astringent and lightly bitter	Dull and lightly dark	78.52
OE8	Smooth	Yellow	Sweet and lightly weak	Coarse and lightly bitter	Even	82.98
OE9	Even color	Lightly dull yellow	Pure	Mellow and lightly astringent	Even and lightly blue leaf	85.65

## Data Availability

The data used to support the findings of this study can be made available by the corresponding author upon request.

## Supplementary Materials

The following supporting information can be downloaded at: https://www.mdpi.com/article/10.3390/foods12234334/s1, Figure S1: Correlation of OE2, Figure S2: Correlation of OE3, Figure S3: VIP plot of OE3, OE4, OE8; Table S1: Catechins contents in fresh leaves, Table S2: Catechins contents under white light withering, Table S3: Catechins contents under yellow light withering, Table S4: Catechins contents under red light withering, Table S5: Catechins contents under blue light withering, Table S6: Catechins contents under different fixing methods, Table S7: Catechins contents under different orthogonal experiment.
