# Supplementary material for: Optimizing Processing Techniques of Oolong Tea Balancing between High Retention of Catechins and Sensory Quality
Source: Foods. 2023 Dec 1;12(23):4334. doi: 10.3390/foods12234334 (PMC10706478; doi:10.3390/foods12234334)
Supplement: Supplementary file 1 [file foods-12-04334-s001.zip › foods-2703022-supplementary.pdf]

Table S1. Catechins' contents in fresh leaves

Compounds	GA	EGC	C	EC	EGCG	GCG	ECG	CG	TC
Fresh Leaves	4.97±0.20	0.10±0.00	10.67±0.34	7.13±0.24	151.25±1.99	12.74±0.38	23.21±0.59	5.78±0.43	210.89±2.99

Table S2. Catechins' contents under white light withering

Compounds	W2	W4	W6	W8	W12	W16	W20	W25
GA	5.24±0.41b	6.49±1.64ab	5.37±0.07b	7.39±0.33a	6.55±0.79ab	6.40±0.23ab	6.19±0.06ab	6.32±0.01ab
EGC	0.14±0.04d	0.19±0.03bcd	0.27±0.02a	0.23±0.05ab	0.20±0.04bc	0.19±0.02bcd	0.18±0.01bcd	0.17±0.02cd
C	11.52±0.96c	12.31±0.59bc	13.08±0.65ab	13.09±0.97ab	13.56±0.10ab	14.13±0.95a	13.06±0.91ab	13.51±0.63ab
EC	6.98±0.42ab	7.01±0.06ab	7.25±0.44a	6.53±0.26abcd	6.07±0.15d	6.34±0.31bcd	6.23±0.40cd	6.81±0.68abc
EGCG	138.29±8.94ab	137.66±3.80ab	140.34±3.15ab	136.29±7.84ab	144.55±2.07a	132.20±7.68b	134.97±2.20ab	134.86±1.36ab
GCG	11.97±0.53cd	11.91±0.48cd	11.68±0.74d	12.03±0.55cd	12.68±0.05bc	13.31±0.64b	12.41±0.22cd	14.38±0.35a
ECG	25.95±0.47ab	26.78±0.18a	25.58±0.75ab	27.68±2.73a	24.37±0.26b	24.20±1.36b	24.03±0.38b	23.77±0.11b
CG	5.53±0.22e	6.02±0.77cde	5.94±0.43de	6.38±0.45bcd	7.01±0.17b	6.81±0.47bc	6.86±0.34bc	7.91±0.53a
TSC	18.64±0.61b	19.52±0.64ab	20.60±0.44a	19.85±0.77ab	19.83±0.19ab	20.66±1.13a	19.47±0.55ab	20.49±1.29a
TETC	181.74±8.32ab	182.38±3.43ab	183.55±3.03ab	182.38±9.68ab	188.61±2.53a	176.51±9.51b	178.27±2.07ab	180.92±1.29ab
TETC/TSC	9.74±0.15a	9.36±0.41abc	8.91±0.18cd	9.19±0.25bc	9.51±0.16ab	8.55±0.08d	9.16±0.16bc	8.86±0.59cd
TC	200.38±8.91a	201.89±3.23a	204.15±3.26a	202.23±10.36a	208.43±2.53a	197.17±10.63a	197.73±2.60a	201.41±0.76a
Bitterness index	9.18±0.19a	8.84±0.41ab	8.47±0.16bcd	8.69±0.25abc	8.98±0.16ab	7.98±0.06d	8.61±0.15bc	8.22±0.55cd

TSC: total simple catechin, TETC: total ester type catechin, TC: total catechin. Values in the same row followed by different lowercase letter are significantly different at $P < 0.05$.

Table S3. Catechins' contents under yellow light withering

Compounds	Y2	Y4	Y6	Y8	Y12	Y16	Y20	Y25
GA	7.76±0.20a	6.22±1.17c	6.55±0.10bc	7.18±0.32ab	7.23±0.27ab	5.92±0.46cd	6.47±0.07bc	5.24±0.20d
EGC	0.15±0.01b	0.21±0.05ab	0.24±0.04a	0.24±0.05a	0.24±0.03a	0.21±0.04ab	0.25±0.02a	0.16±0.06b
C	12.13±0.31c	11.85±0.34c	13.13±1.32bc	12.69±1.63c	14.56±0.48ab	14.51±0.34ab	15.10±0.53a	12.08±0.39c
EC	6.74±0.27abc	7.09±0.30ab	7.34±0.15a	6.86±0.55abc	6.44±0.20bc	6.35±0.19c	6.32±0.15c	7.13±0.63a
EGCG	124.00±3.93c	146.87±1.05a	128.09±4.16bc	132.07±4.51bc	128.88±7.90bc	134.93±5.24bc	129.84±0.97bc	123.93±4.44c
GCG	13.31±0.28bc	12.53±0.17bc	13.26±1.25bc	12.43±0.21c	13.52±0.27b	13.48±0.54b	15.26±0.27a	13.01±0.46bc
ECG	25.34±0.72bc	29.49±2.62a	24.20±0.18bc	23.38±0.75c	24.56±0.38bc	24.43±1.04bc	26.33±0.84b	26.12±2.13b
CG	6.89±0.37bc	6.45±0.37c	6.79±0.54c	6.42±0.49c	7.61±0.07b	7.01±0.48bc	8.45±0.46a	6.48±0.37c
TSC	19.02±0.41c	19.14±0.51c	20.72±1.13ab	19.79±1.17bc	21.23±0.70a	21.07±0.32ab	21.67±0.39a	19.38±0.63c
TETC	169.54±5.16b	195.34±2.78a	172.33±4.41b	174.30±5.47b	174.56±8.52b	179.85±7.24b	179.88±0.52b	169.54±6.61b
TETC/TSC	8.92±0.35b	10.21±0.32a	8.33±0.49bc	8.82±0.39bc	8.22±0.23c	8.54±0.24bc	8.30±0.15c	8.75±0.19bc
TC	188.56±5.11c	214.48±2.78a	193.05±4.56bc	194.09±6.35bc	195.79±9.11bc	200.92±7.53b	201.55±0.69b	188.91±7.13c
Bitterness index	8.29±0.33b	9.67±0.29a	7.79±0.42bc	8.30±0.35b	7.68±0.23c	7.99±0.24bc	7.70±0.15c	8.15±0.21bc

TSC: total simple catechin, TETC: total ester type catechin, TC: total catechin. Values in the same row followed by different lowercase letter are significantly different at $P < 0.05$.

Table S4. Catechins' contents under red light withering

Compounds	R2	R4	R6	R8	R12	R16	R20	R25
GA	8.10±0.34a	7.18±0.26abc	5.92±1.60d	7.56±0.32ab	6.60±0.07bcd	6.27±0.05cd	5.80±0.16d	5.95±0.24d
EGC	0.14±0.02c	0.22±0.03bcd	0.28±0.04a	0.23±0.04ab	0.23±0.02ab	0.19±0.05bc	0.19±0.05bc	0.18±0.01bc
C	11.75±0.43b	12.67±0.22ab	13.00±1.55ab	13.47±1.28ab	13.29±0.95ab	12.58±0.51b	13.62±1.32ab	14.78±1.62a
EC	6.95±0.22ab	7.04±0.06a	6.71±0.21abc	7.11±0.15a	6.40±0.22bcd	6.15±0.42cd	6.26±0.56cd	6.09±0.38d
EGCG	133.80±4.54c	137.04±2.42bc	144.83±4.15a	144.97±1.87a	138.19±2.93bc	119.00±4.00d	140.85±4.80ab	134.90±0.66bc
GCG	12.40±0.34bc	11.23±0.28d	11.73±0.85cd	12.29±0.12bc	11.50±0.20d	13.22±0.23a	12.42±0.33bc	12.50±0.38b
ECG	26.52±0.37b	23.33±0.76d	25.81±1.38bc	30.58±1.38a	24.42±2.21bcd	22.79±0.43d	25.75±0.97bc	23.98±0.68cd
CG	6.52±0.17ab	6.69±0.31ab	6.59±0.73ab	6.60±0.43ab	6.03±0.21b	6.53±0.10ab	6.51±0.34ab	7.02±0.56a
TSC	18.84±0.24c	19.92±0.29abc	19.99±1.67abc	20.81±1.28ab	19.93±0.74abc	18.92±0.71bc	20.07±0.83abc	21.05±1.24a
TETC	179.25±5.35c	178.30±2.53c	188.97±6.89ab	194.44±1.93a	180.13±5.30c	161.54±4.48d	185.54±4.87bc	178.40±1.14c
TETC/TSC	9.51±0.16a	8.95±0.14ab	9.48±0.48a	9.37±0.51a	9.04±0.24ab	8.54±0.15b	9.25±0.14a	8.49±0.47b
TC	198.10±5.58c	198.22±2.65c	208.96±8.53ab	215.25±2.98a	200.06±5.86bc	180.45±5.14d	205.61±5.70bc	199.46±2.20bc
Bitterness index	8.93±0.16a	8.49±0.13ab	9.04±0.51a	8.89±0.47a	8.58±0.22a	7.93±0.16b	8.72±0.14a	7.98±0.47b

TSC: total simple catechin, TETC: total ester type catechin, TC: total catechin. Values in the same row followed by different lowercase letter are significantly different at $P < 0.05$.

Table S5. Catechins' contents under blue light withering

Compounds	B2	B4	B6	B8	B12	B16	B20	B25
GA	7.99±0.20a	7.81±0.43ab	7.09±0.30c	7.34±0.32bc	6.11±0.29de	6.56±0.06d	5.92±0.11e	5.40±0.36f
EGC	0.15±0.02c	0.26±0.03a	0.28±0.04a	0.26±0.02a	0.24±0.01ab	0.22±0.05ab	0.26±0.02a	0.19±0.06bc
C	12.44±0.70d	13.41±1.35cd	13.71±0.58bcd	14.94±0.85ab	15.08±0.39ab	15.74±0.54a	15.24±0.27a	14.56±0.91abc
EC	6.70±0.04b	7.86±0.51a	8.21±0.09a	6.08±0.13c	6.15±0.25c	6.40±0.04bc	6.29±0.21bc	6.47±0.14bc
EGCG	134.57±11.36b	139.06±8.18ab	148.04±0.18a	142.87±2.58ab	146.74±3.19a	141.78±4.45ab	139.58±1.18ab	137.76±0.74ab
GCG	12.07±0.27cd	12.79±0.61bc	12.50±0.40bcd	11.74±0.84cd	11.54±0.53d	14.52±0.12a	12.33±0.43bcd	13.31±0.84b
ECG	26.73±0.54bc	29.89±0.77a	28.93±0.32ab	29.70±2.14a	27.63±0.78abc	25.67±0.10cd	23.69±0.96d	24.03±2.63d
CG	6.11±0.49b	6.73±0.49b	6.56±0.25b	6.12±0.70b	5.82±0.49b	7.63±0.06a	6.49±0.44b	8.07±0.66a
TSC	19.29±0.67b	21.53±0.83a	22.19±0.65a	21.28±0.73a	21.47±0.15a	22.37±0.50a	21.79±0.23a	21.22±1.03a
TETC	179.47±12.53c	188.47±7.25abc	196.03±0.50a	190.43±5.63abc	191.73±3.30ab	189.59±4.60abc	182.08±0.67bc	183.17±3.15bc
TETC/TSC	9.30±0.35a	8.77±0.67ab	8.84±0.24ab	8.96±0.54ab	8.93±0.20ab	8.48±0.02b	8.36±0.07b	8.65±0.52ab
TC	198.77±13.18c	210.00±6.43ab	218.22±1.14a	211.71±5.12ab	213.19±3.20ab	211.95±5.11ab	203.87±0.85bc	204.39±2.80bc
Bitterness index	8.75±0.37a	8.29±0.69ab	8.39±0.23ab	8.52±0.50ab	8.50±0.18ab	7.92±0.04b	7.90±0.08b	8.10±0.45ab

TSC: total simple catechin, TETC: total ester type catechin, TC: total catechin. Values in the same row followed by different lowercase letter are significantly different at $P < 0.05$.

Table S6. Catechins' contents under different fixing methods

Treatment	Compound												
	GA	EGC	C	EC	EGCG	GCG	ECG	CG	TSC	TETC	TETC /TSC	TC	Bitterness index
F1	10.20±0.27d	0.19±0.01c	4.32±0.16bc	4.58±0.29def	117.84±3.64d	17.10±0.65a	31.44±0.75abc	7.50±0.34h	9.09±0.34cd	173.87±4.19ef	19.15±1.03f	182.96±4.00e	17.66±1.06f
F2	8.53±0.34f	0.16±0.00f	2.55±0.22g	6.33±0.60a	102.05±1.19e	14.68±0.72b	28.42±0.40e	8.62±0.22efg	9.03±0.40cd	153.77±0.74g	17.04±0.76g	162.81±0.86f	15.71±0.80g
F3	9.88±0.34d	0.23±0.01a	3.09±0.08ef	4.50±0.11ef	149.21±0.83a	11.04±0.54def	26.57±0.53fg	7.58±0.14h	7.82±0.20f	194.40±0.39bc	24.87±0.58c	202.22±0.56c	24.18±0.64bc
F4	7.34±0.12g	0.14±0.01g	5.90±0.66a	4.74±0.23bcde	141.24±0.91b	10.41±0.02f	31.57±0.56ab	8.59±0.28efg	10.78±0.69b	191.81±0.64c	17.84±1.21fg	202.60±0.46c	17.12±1.18fg
F5	9.23±0.19e	0.19±0.01c	4.39±0.22bc	4.98±0.23bcd	149.91±1.66a	11.74±0.58d	31.40±0.38abc	10.20±0.19ab	9.57±0.40c	203.26±2.57a	21.27±0.69e	212.82±2.90a	20.47±0.71e
F6	8.65±0.14f	0.16±0.00f	6.23±0.22a	5.05±0.07bc	140.47±1.67b	11.26±0.55def	32.16±0.36a	8.09±0.15gh	11.44±0.30a	191.99±2.55c	16.79±0.22g	203.43±2.85bc	16.05±0.25fg
F7	8.48±0.29f	0.16±0.00ef	4.55±0.10b	3.08±0.17h	131.07±1.45c	10.39±0.51f	25.99±0.52g	7.48±0.14h	7.79±0.10f	174.93±2.50ef	22.45±0.60e	182.73±2.40e	21.59±0.55e
F8	8.61±0.29f	0.17±0.00de	3.55±0.11d	2.87±0.07hi	129.08±1.43c	10.59±0.52ef	25.79±0.52g	7.45±0.14h	6.59±0.07g	172.92±2.48f	26.23±0.34bc	179.51±2.52e	25.31±0.28b
F9	7.34±0.20g	0.13±0.01h	3.17±0.34def	2.52±0.13i	129.22±4.31c	10.78±0.20def	30.25±0.53cde	8.98±0.77def	5.82±0.40h	179.23±4.80e	30.89±2.66a	185.06±4.57e	29.73±2.62a
F10	11.82±0.27a	0.21±0.00b	4.02±0.11c	4.70±0.18cde	141.10±0.69b	11.46±0.24de	30.61±0.34bcd	9.64±0.52bc	8.93±0.17de	192.81±0.45c	21.61±0.43e	201.73±0.44c	20.83±0.41e
F11	10.80±0.18c	0.21±0.00b	4.01±0.11c	4.16±0.18f	147.56±1.63a	10.80±0.23def	25.38±0.51g	8.42±0.15fg	8.38±0.07e	192.16±2.47c	22.94±0.39de	200.54±2.45c	22.21±0.38de
F12	11.86±0.20a	0.19±0.00c	3.41±0.09de	5.17±0.20b	149.67±1.66a	11.46±0.24de	28.37±0.57de	10.30±0.19a	8.77±0.27de	199.79±2.59ab	22.79±0.72e	208.57±2.66ab	21.99±0.71de

F13	11.35±0.19b	0.12±0.01i	4.07±0.11c	3.56±0.21g	138.93±1.54b	11.49±0.57de	29.96±0.60d	9.55±0.17cd	7.74±0.33f	189.93±2.74cd	24.54±0.77cd	197.68±3.02cd	23.43±0.79cd
F14	11.73±0.20ab	0.17±0.00d	3.60±0.10d	3.19±0.05gh	133.10±1.47c	13.16±0.65c	30.06±0.60d	9.13±0.17cde	6.96±0.15g	185.45±2.75d	26.66±0.29b	192.41±2.88d	25.43±0.34b
F15	6.68±0.27h	0.14±0.01g	2.81±0.13fg	3.11±0.35h	130.63±6.07c	11.07±0.60def	27.62±1.68ef	8.36±0.77fg	6.06±0.22h	177.68±8.12ef	29.34±1.12a	183.74±8.24e	28.19±1.26a

TSC: total simple catechin, TETC: total ester type catechin, TC: total catechin. Values in the same column followed by different lowercase letter are significantly different at $P < 0.05$.

Table S7. Catechins' contents under different orthogonal experiment

Compound	CK	OE1	OE2	OE3	OE4	OE5	OE6	OE7	OE8	OE9
GA	11.39±0.37b	9.41±0.18d	8.21±0.17e	12.41±0.43a	11.55±0.27b	9.62±0.24cd	8.21±0.13e	9.52±0.34d	11.75±0.11b	10.01±0.11c
EGC	0.18±0.01bc	0.17±0.00cde	0.10±0.02g	0.15±0.01de	0.15±0.00e	0.20±0.00a	0.11±0.00g	0.19±0.02ab	0.13±0.00f	0.17±0.00bcd
C	10.72±0.13a	10.25±0.08b	10.11±0.25b	9.57±0.32c	10.25±0.15b	9.28±0.23c	10.24±0.21b	10.26±0.24b	9.51±0.22c	9.35±0.33c
EC	22.84±0.28c	20.17±0.37d	14.23±0.18f	25.68±0.66b	26.96±0.94a	22.84±0.17c	16.39±0.30e	19.21±0.94d	23.43±0.18c	19.47±0.54d
EGCG	114.79±0.86g	119.62±2.93f	113.76±2.45g	147.11±1.40a	139.12±3.04b	125.25±1.68e	119.17±0.24f	135.00±3.36c	131.16±0.58d	138.67±1.61b
GCG	19.54±0.43d	17.98±0.20f	15.01±0.40h	28.08±0.21a	23.75±0.58c	18.99±0.23de	14.48±0.27h	16.86±0.51g	25.57±0.55b	18.78±0.48e
ECG	31.48±1.56cd	30.31±0.86de	24.20±0.72g	36.61±0.44a	33.59±1.33b	29.35±0.34e	30.75±0.63de	27.40±1.01f	32.54±0.65bc	26.40±0.43f
CG	5.06±0.21c	4.33±0.35e	6.18±0.67a	5.87±0.05ab	5.22±0.21c	4.30±0.21e	2.98±0.06f	5.03±0.45c	6.06±0.13a	5.37±0.33bc
TSC	33.74±0.20c	30.59±0.40e	24.44±0.43h	35.41±0.44b	37.36±1.07a	32.31±0.36d	26.74±0.49g	29.66±0.87ef	33.07±0.05cd	28.99±0.34f
TETC	170.86±2.80g	172.24±3.41g	159.16±2.92h	217.66±1.82a	201.68±4.98b	177.89±2.27f	167.38±0.66g	184.29±3.28e	195.32±1.84c	189.22±2.54d
TETC/TSC	5.06±0.06f	5.63±0.04d	6.51±0.14a	6.15±0.03b	5.40±0.11e	5.51±0.10de	6.26±0.13b	6.22±0.11b	5.91±0.07c	6.53±0.02a
TC	204.60±2.98f	202.83±3.81f	183.60±3.08h	253.07±2.26a	239.04±5.78b	210.20±2.19e	194.13±0.62g	213.95±4.03de	228.39±1.79c	218.21±2.88d
Bitterness index	4.52±0.05g	5.08±0.04de	5.93±0.12a	5.38±0.02c	4.79±0.10f	4.95±0.09e	5.75±0.12b	5.69±0.11b	5.16±0.05d	5.92±0.02a

TSC: total simple catechin, TETC: total ester type catechin, TC: total catechin. Values in the same row followed by different lowercase letter are significantly different at $P < 0.05$.

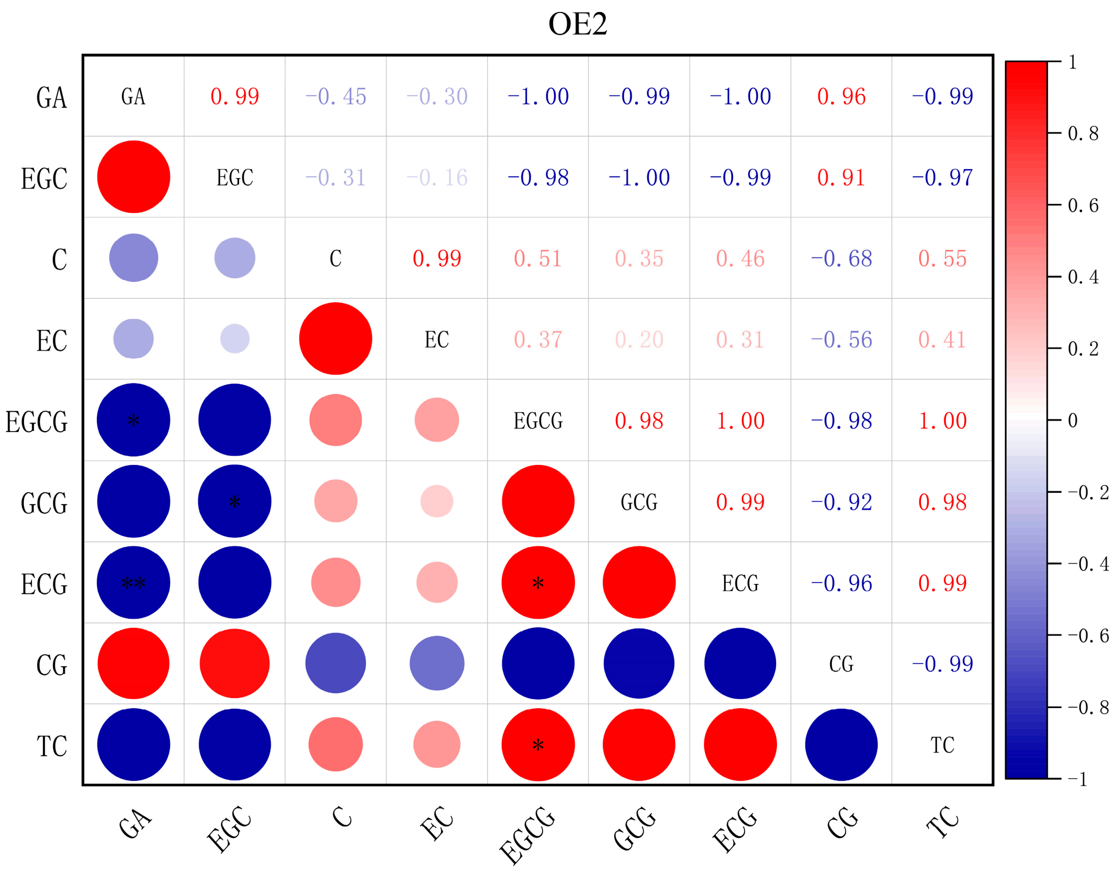

Figure S1. Correlation of OE2

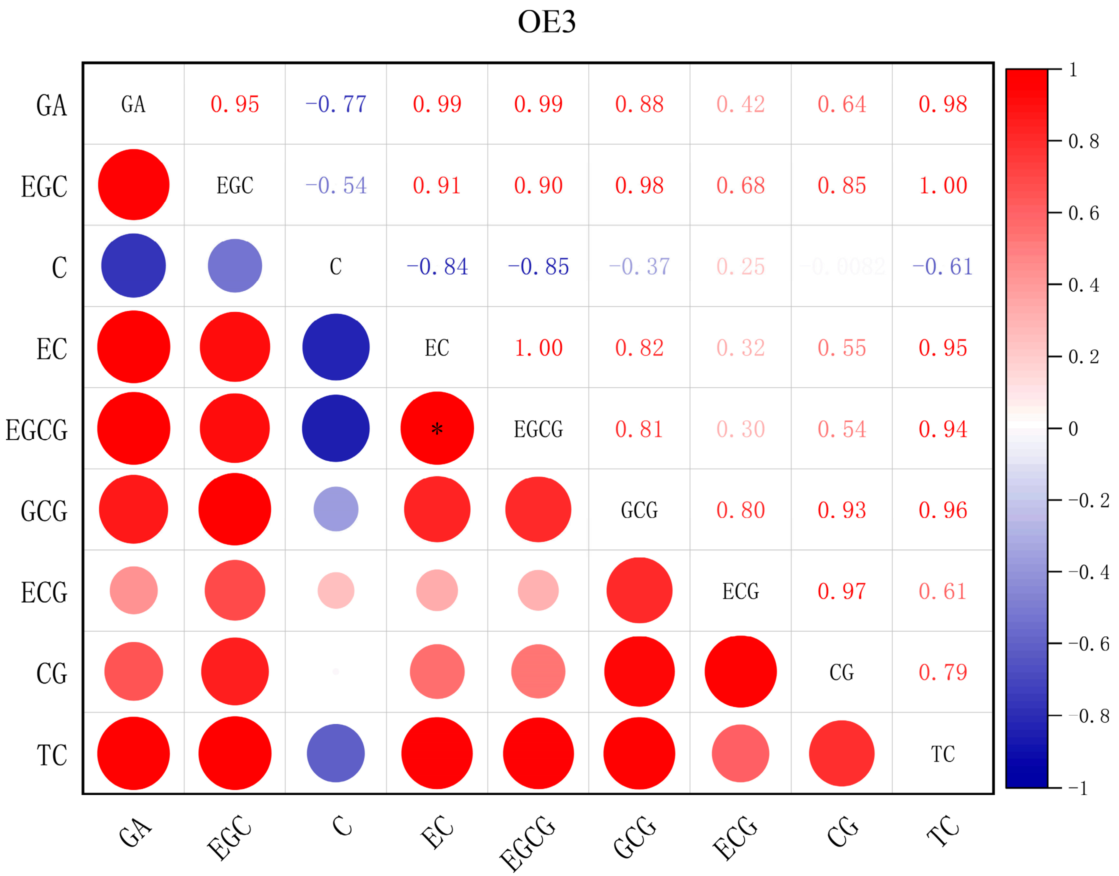

Figure S2. Correlation of OE3

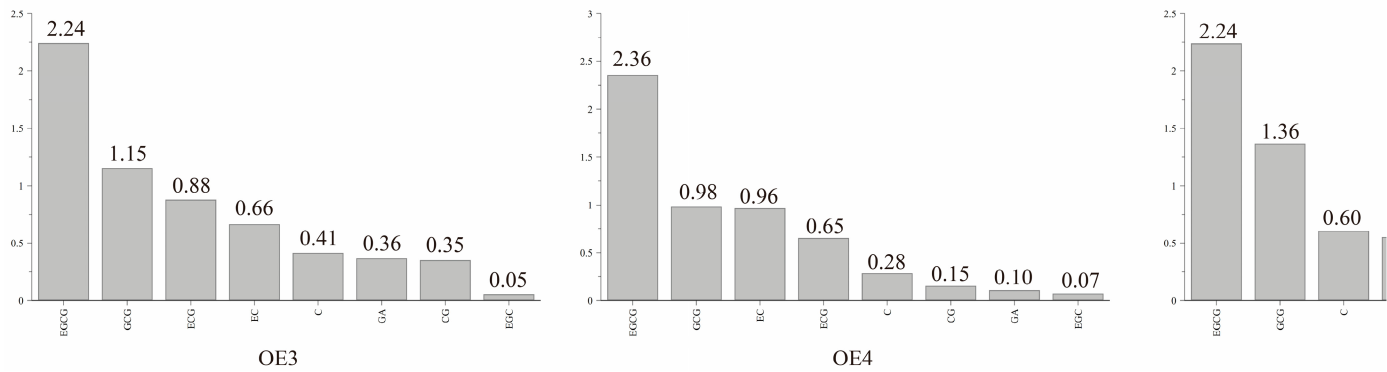

Figure S3. VIP plot of OE3, OE4, OE8
